# Evaluating the effectiveness of an empowerment program for self-care in type 2 diabetes: a cluster randomized trial

**DOI:** 10.1186/s12889-016-3937-5

**Published:** 2017-01-06

**Authors:** Daniel Nogueira Cortez, Maísa Mara Lopes Macedo, Débora Aparecida Silva Souza, Jéssica Caroline dos Santos, Gesana Sousa Afonso, Ilka Afonso Reis, Heloísa de Carvalho Torres

**Affiliations:** 1 Federal University of São João del-Rei (Centro Oeste Campus), Divinópolis, Brasil; 2 School of Nursing, Federal University of Minas Gerais, Belo Horizonte, Brazil; 3 Institute of Exact Sciences, Federal University of Minas Gerais, Belo Horizonte, Brazil; 4 Universidade Federal de São João Del-Rei, Sebastião Gonçalves Coelho Street, 400, sala 302.1D, Divinópolis, MG ZIP CODE: 35.501-296 Brazil

**Keywords:** Diabetes type 2, Health education, Randomized controlled trial, Self care, Primary health care

## Abstract

**Background:**

The prevalence of type 2 diabetes mellitus is increasing substantially worldwide, leading to serious economic effects, complications and deaths. This study evaluated the effectiveness of an empowerment program providing support for psychosocial, behavioral, and clinical aspects of diabetes to help Brazilian users of public health services obtain metabolic control of this condition.

**Methods:**

In this cluster randomized trial, participants aged 30–80 diagnosed with type 2 diabetes were recruited from ten Brazilian public health units in 2014 and 2015. Five units were randomly assigned to receive the empowerment program based on a behavior change protocol, and five continued to receive only conventional treatment. The primary outcome was the biochemical and anthropometric parameters, and the secondary outcomes were self-care, attitude, knowledge and empowerment related to diabetes. The effect of the experiment was defined as the percentage variation between the values at the initial and final periods. To evaluate this effect and to compare it in the two groups, tests were used for paired and independent samples, respectively.

**Results:**

There were 238 participants: 127 and 111 in the intervention and control group, respectively. For glycated hemoglobin, the mean effect in the control and intervention groups was 3.93 and −5.13, respectively (*p* < 0.001). Levels of glycated hemoglobin and other metabolic indicators, as well as the most part of the secondary outcomes showed a significant difference in the experimental group compared to the control group.

**Conclusions:**

The empowerment program improved metabolic control of type 2 diabetes in Brazilian users.

**Trial registration:**

NCT02132338 - April 22, 2014.

## Background

Diabetes mellitus has a high prevalence worldwide, leading to problems such as increased mortality and health costs [1, 2]. There were 387 million cases of diabetes in 2014, and 415 million in 2015. It is predicted that there will be 642 million cases in adults between 20 and 79 years of age by 2040. In 2015 alone, five million died from diabetes [3, 4]. Brazil is the country with the fourth-largest number of people with diabetes: 14.3 million; 130.000 people died from this condition in Brazil, more than in any other country in Central and South America, and more than half of all diabetes deaths in the region in 2013 [4].

Brazil has a public policy of following up users with DM through the primary healthcare service [5]. The Ministry of Health implemented the DM characterization measures through a national study that traced the health status of users as well as expansion of the public health teams and their training for the care of several chronic conditions and interventions to change specific behavior for DM [5, 6].

To reduce the impact of diabetes in Brazil, proactive, integrative, interdisciplinary and continuous educational programs need to be developed, providing individualized care for each person based on the context of their life [5]. Traditional methods of care alone, which utilize prescriptive actions the patient is expected to adhere to in order to control diabetes, are unable to stop the progression of this condition [6, 7].

Educational programs based on empowerment use a participatory process that allows people with diabetes to be responsible for their own condition, sharing that responsibility with health care professionals, and having their actions in care management acknowledged [8, 9]. Patients who indicate they can make their own decisions are more likely to take responsibility for their own diabetes care [5, 10], and many studies around the world have addressed this approach [8, 11–14].

From this perspective, an empowerment program based on dialog, exchange, and Freirean theories was developed in a Brazilian city [15]; this program involved behavioral, psychosocial, and clinical aspects related to diabetes in an effort to control this condition. Thus, the objective of this study was to evaluate the effectiveness of an empowerment program for metabolic control aimed at Brazilian patients in the public health system with type 2 diabetes.

## Methods

This randomized cluster trial involved public health users with type 2 diabetes who received services from 10 primary care units in one Brazilian town from December 2014 to December 2015. The study was approved by the Research Ethics Committee of the Federal University of Minas Gerais, Brasil, under Process 426.968/2013, and all participants signed an informed consent agreement. The study is listed in the International Clinical Trials as NCT02132338 and in the Brazilian registry as RBR-92j38t.

### Participants

The study involved users of public health services who met the eligibility criteria for random distribution into the study groups. The following criteria were required: having type 2 diabetes, being literate, aged between 30 and 80 years, having no serious complications, being open to communication and cooperation, agreeing to attend group meetings at the health units and to receive visits at home, providing information for telephone contact, and being sufficiently independent to perform self-care activities.

Based on a study involving the population of users with diabetes in the town [16] and data from a previous study [17] by the research group, the sample size was calculated considering the cluster effect [18]. The calculation has resulted in 100 participants for each group (control and intervention group). Using R software (R Core Team, 2015), various combinations of the ten units were composed and allocated to two groups of five units each. One of the combinations that satisfied the homogeneity criteria for these groups with regard to age, glycated hemoglobin, and education level was randomly selected. Next, one of the groups was randomly allocated to receive the intervention (IG) and the other was allocated as the control group (CG).

### Sample size calculation

To calculate the unadjusted sample size (*m*) in each group, which does not consider the clustering effect, we have modified the expression (2) reported by Campbell et al. (2004) [18] considering a finite population as follows:

$$ m=\frac{\left(\raisebox{1ex}{$N$}\!\left/ \!\raisebox{-1ex}{$2$}\right.\right)\times 4{\left({z}_{1-\left(\alpha /2\right)}+{z}_{\varpi}\right)}^2}{\left(\raisebox{1ex}{$N$}\!\left/ \!\raisebox{-1ex}{$2$}\right.-1\right){d}^2+4{\left({z}_{1-\left(\alpha /2\right)}+{z}_{\varpi}\right)}^2}, $$ where *d* is the anticipated standardized effect size for the main outcome [glycosylated hemoglobin (HbA1c), %], i.e., the minimum difference to be detected between two groups and is termed as standard deviation; α is the tolerable Type I error rate (significance level in the hypothesis tests); ω is the desired level of statistical power (the probability of rejecting the null hypothesis given that it is false); $$ \overline{n} $$ is the average cluster size; ρ is the intra-class correlation (ICC) coefficient, which measures the degree of similarity among individuals inside the clusters; *k* is the number of clusters (basic health units); N is the total population; and Z_1-α/2_ and Z_ω_ are the standard normal percentiles.

To accommodate the clustering effect, an inflation factor was calculated using the equation $DE=1+\left(\overline{n}-1\right)\rho$, which is also commonly known as the “design effect” [18]. This procedure increases the sample size to account for the homogeneity of the individuals inside clusters larger than that expected in a design without clusters. To obtain the adjusted sample size (*n*) in each group (control and intervention), the unadjusted sample size (*m*) was multiplied using the design effect (*DE*).

The values used in sample size calculation were α = 0.05, ω = 0.90, *d* = 1, $\overline{n}$ = 80.9, and *N* = 1320. The value for ρ was estimated using the data of a previous project involving users diagnosed with Type 2 Diabetes in a city near Divinopolis [17]. From these data, we obtained the value ρ = 0.008. Then, we calculated *n* = 65 users for each study group. Considering a participant attrition rate of 35%, we should have, at least, 100 users in each group.

### Intervention

The entire program was based on the behavior change protocol that was validated for Brazil [19, 20]. The steps contained in the protocol are explore the problem, identify and discuss feelings and meanings, set goals, create a care plan to achieve the goal(s), and evaluate the experience and the plan. Together, these elements were designed to facilitate and produce effective interactions between health professionals and users.

**Fig. 1:**
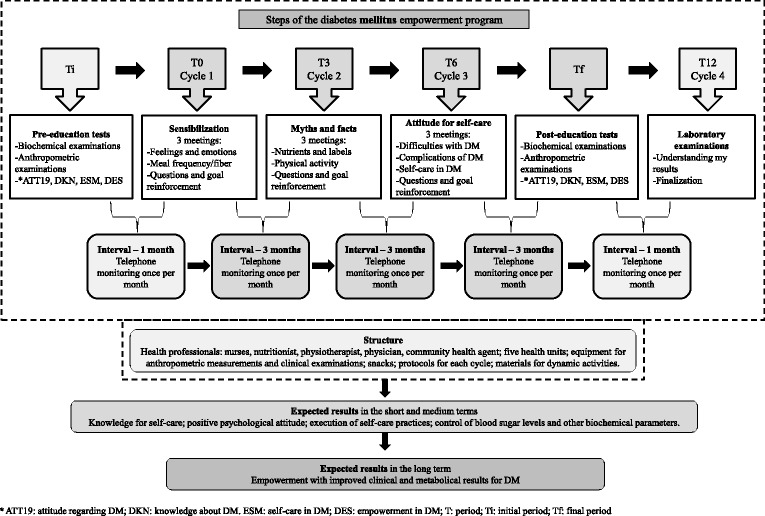
Model of the empowerment program for self-care in diabetes mellitus

The intervention took place over 12 months; this was divided into the initial period when the pre-education tests occurred (Ti), period 0 when cycle 1 occurred (T0), period 3 when cycle 2 occurred (T3), period 6 when cycle 3 occurred (T6), period 12 when cycle 4 occurred (T12), and the final period when the post-education tests took place (Tf). Each cycle lasted three months. Therefore, program unfolded over six distinct periods, as seen in the model (Fig. 1).

There were 10 meetings of IG over 12 months, as described in Fig. 1; the meetings lasted an average of 2 h each. The meetings were divided into four cycles; the first to the third cycle had three meetings at seven-day intervals and three months between each cycle, and the fourth cycle was performed in one last meeting to deliver test results and to complete the educational program. During the periods between cycles, the users were contacted monthly by telephone to be monitored with regard to questions and motivated about the behavior change goals that were proposed in the meetings. CG did not attend the educational meetings, but similar to IG, CG received the same routine care from the health teams (conventional monitoring performed in the Basic Health Units through clinical care).

Each health unit of IG was divided into groups with a maximum of 10 participants, and the meetings of these groups were held at a variety of different times and days so that participants could choose according to their availability resulting from work or other activities. It is important to note that each IG comprised members of the same team to avoid contamination. The search for dialog and exchange of experiences in the interventions was based on Freirean theory [15]. Home visits were made when users were absent from group meetings, as indicated in the protocol, in order to encourage them to return and participate.

The first cycle, T0, was used as a baseline for the other cycles, as it entailed three weeks of intense discussions about the feelings and meanings of diabetes mellitus throughout the lives of the participants, identifying their needs and building the foundation for empowerment. At the end of the initial cycle, respecting the protocol and not neglecting work with feelings and meanings, it was possible to construct the working dynamics for the other cycles using the main topics listed by the users: physical activity (types, frequency, stretching, proper footwear, obesity), nutritional reeducation (frequency, amount, composition of foods, fiber, liquids, labels), quality of life (anxiety, stress, relaxation, influence on control of DM) complications of DM (types, care), self-care (managing your life, valuing your time, evaluating your choices and consequences) and empowerment (goals for each cycle).

The lead researcher guided the work in the meetings as a facilitator and an instigator of discussions, with the support of at least one more assistant. The problem situations that were identified were clarified by the participants, based on the life experience of those involved. At the end of each meeting, each user set a goal to be achieved as a way to respond to the problem that was addressed.

### Measurements

To collect data on secondary outcomes, four questionnaires involving diabetes mellitus and validated for Brazil were used to collect the data.

The questionnaire that evaluates knowledge (DKN) [21] contains 15 multiple-choice questions about different aspects related to general knowledge about diabetes. It addresses hypoglycemia, food groups and their replacements, cautions for diabetes complications, and general principles of this condition. Its score can range from 1–15 points.

The questionnaire about user attitudes (ATT) [21] is a measure of psychological adjustment for diabetes containing 19 questions. Its score can range from 19–95 points.

The self-care questionnaire (ESM) [22] measures adherence to self-care activities in users with diabetes, and contains eight questions. Its score can range from 1–8 points.

The short form empowerment scale (DES) [23] was designed to assess psychosocial self-sufficiency in diabetes and contains eight items that cover the following areas: need for change, developing a plan, overcoming obstacles, support, dealing with emotions, self-motivation, and making care choices for diabetes that are appropriate for the individual's own priorities and circumstances [8]. Its score can range from 1–5 points.

These instruments were applied to all the study participants at two different times: at the beginning of the study, before any educational activity, and at the end of the study, comparing the results for Tf and Ti.

Questionnaires were also applied to collect demographic data such as age, sex, education level, occupation, and marital status, and measurements were taken for systolic blood pressure (SBP) (mmHg), diastolic blood pressure (DBP) (mmHg), BMI (km/m^2^), and waist circumference (WC) (cm). The measures of SBP/DBP, BMI (weight and height), and WC were obtained twice during each data collection to obtain the mean value in a way that reduces measurement errors. Blood was collected to measure HbA1c (%), triglycerides (TGL) (mg/dl), total cholesterol (TC) (mg/dl), light density lipoprotein (LDL) (mg/dl), and high density lipoprotein (HDL) (mg/dl) [2]. The examinations were performed at a single laboratory both at baseline and the end period of the study. HbA1c levels were measured using high-performance liquid chromatography and lipid levels were measured using enzymatic colorimetry.

Since the results were directly compared both between groups and within each group, a cutoff value was not established for normality in the parameters used in the study.

### Analysis of the data

Statistical Package for the Social Sciences® (SPSS) software version 20.0 was used to carry out the descriptive analysis, along with frequency calculation and measures of central tendency and dispersion. The Shapiro–Wilk normality test was applied to verify the distribution of continuous quantitative variables.

To investigate differences in categorical variables between the study groups at baseline, logistic regression models were used considering a cluster factor nested to the group factor. For all quantitative variables, the effect of the experiment on a variable was defined as the difference between its values in the final period and initial period (Δ) divided by the initial value. The values of the effect were multiplied by 100 to transform them in percentage variations. To assess the difference in this effect between the experimental groups as well as to compare the groups at baseline, a nested ANOVA was used (cluster factor nested into group factor to account for the clusters randomization). To assess the effectiveness of the intervention within the groups, a block factor (individuals) was added to the model, the *p*-values for the F tests of ANOVA are calculated by Monte Carlo simulation the ANOVA model, since these types of response variables hardly follow the normal distribution [24]. The simulation experiments were carried out in the R statistical programming environment as well as the logistic regression analysis [25]. For all analyses, a 95% confidence level was used (*p* < 0.05).

## Results

**Fig. 2:**
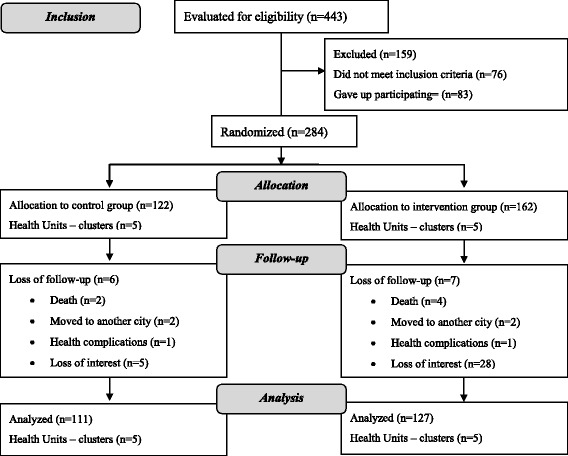
Diagram of the progress of clusters and individuals in the phases of the randomized trial

Following the guidelines of CONSORT [26], Fig. 2 shows a flow diagram of progress of the clusters and individuals through phases of the randomized trial. After randomization, IG contained 162 users, and CG included 122 users. After losses, 238 users with type 2 diabetes participated in the study analysis: 127 in IG and 111 in CG. In all, the study had a 16% sample loss. For both CG and IG, there was no statistically significant difference for the variables sex, age, education level, body mass index (BMI), and glycated hemoglobin (HbA1c) between those users who left and those who remained in the study (*p* > 0.05, Tables 3 and 4 in Appendix).

The mean age of the study population was 57.8 years ± 9.43 standard deviations (SD). The results of this study showed that users in CG and IGs were homogeneous at baseline with regard to the socio-demographic variables (Table 1), biochemical and anthropometric variables, and secondary outcomes (Table 2). The results also showed that the users (in control and intervention groups) showed moderate glycemic control, and the mean HbA1c for the 238 participants was 7.98% ± 1.95 (SD) in the initial period.

**Table 1:** Descriptive statistics for socio-demographic variables of users with type 2 diabetes

Variable	Mean ± SD or N (%)		p*
	CG (*n* = 111)	IG (*n* = 127)	
Age (Mean ± SD)	57.5 ± 9.7	58 ± 9.2	0.615
Sex (N (%))			
Male	38 (34.2)	42 (33.1)	0.880
Female	73 (65.8)	85 (66.9)	
Education Level (N (%))			
Incomplete elementary school	73 (65.8)	90 (70.9)	0.399
Elementary school complete to post-graduate	38 (34.2)	37 (27.1)	
Marital Status (N (%))			
With companion	87 (78.4)	94 (74)	0.432
Without companion	24 (21.6)	33 (26)	
Occupation (N (%))			
Active	55 (49.5)	55 (43.3)	0.336
Inactive	56 (50.5)	72 (56.7)	

*Linear or logistic regression

*CG* Control group, *IG* Intervention group

**Table 2:** Comparison of the groups for anthropometric indicators and indicators of metabolic control, and secondary outcomes at baseline and after intervention

Variables	CG			IG			CG-IG (baseline)
	Initial Time Mean ± SD	Final Time Mean ± SD	p*	Pre-education Mean ± SD	Post-education Mean ± SD	p*	p**
HbA1c	7.9 ± 1.9	8.1 ± 2.2	0.117	8.1 ± 2.0	7.5 ± 1.7	<0.001	0.632
TC	191.9 ± 39.5	180.8 ± 45.0	<0.001	187.4 ± 39.7	171.5 ± 39.2	<0.001	0.591
HDL	41.7 ± 11.3	47.5 ± 12.5	<0.001	43.9 ± 10.3	46.2 ± 11.3	<0.001	0.148
LDL	113.8 ± 35.8	95.9 ± 36.8	0.492	105.1 ± 32.9	89.6 ± 32.4	<0.001	0.278
VLDL	36.4 ± 22.3	37.9 ± 30.4	<0.001	38.7 ± 23.7	36.1 ± 21.7	0.069	0.335
TGL	180.2 ± 110.8	189.4 ± 152.0	0.409	192.7 ± 119.3	180.8 ± 108.6	0.092	0.251
BMI	30.0 ± 6.0	29.9 ± 5.9	0.393	30.7 ± 5.8	30.5 ± 5.5	0.345	0.445
SBP	132.1 ± 16.2	135.2 ± 19.5	0.097	130.0 ± 18.3	129.1 ± 18.3	0.642	0.249
DBP	82.8 ± 13.0	83.6 ± 12.0	0.531	82.4 ± 11.9	79.3 ± 10.1	0.024	0.827
WC	96.0 ± 12.9	96.1 ± 12.9	0.197	98.2 ± 11.4	98.3 ± 11.4	0.226	0.252
SLC	3.9 ± 1.2	3.3 ± 1.1	0.820	3.4 ± 1.1	4.2 ± 1.2	<0.001	0.619
KNW	9.0 ± 2.1	9.2 ± 2.6	0.366	9.2 ± 2.3	12.6 ± 1.7	<0.001	0.603
ATT	63.1 ± 10.7	68.4 ± 11.0	<0.001	61.9 ± 9.4	78.2 ± 11.9	<0.001	0.270
EPW	3.7 ± 0.4	3.9 ± 0.5	<0.001	3.7 ± 0.4	4.1 ± 0.4	<0.001	0.817

* Nested ANOVA using a block factor for comparison before and after intra group

** Nested ANOVA for comparison between groups at baseline

*CG* Control Group, *IG* Intervention group, *HbA1c* Glycated hemoglobin, *TC* Total Cholesterol, *HDL* High density lipoprotein, *LDL* Low density lipoprotein, *TGL* Triglycerides, *BMI* Body mass index, *SBP* and *DBP* Systolic and diastolic blood pressure, *WC* Waist circumference, *SLC* Self-care for DM2, *KNW* Knowledge for DM2, *ATT* Attitude for DM2, *EPW* Empowerment for DM2

With respect to the biochemical and anthropometric parameters, it was observed that among the participants in IG, after intervention there was a statistically significant reduction in values for HbA1c, TC, and LDL and DBP, and an increase in HDL (*p* < 0.05). Among the individuals in CG, in turn, after the end of the study there was a reduction in TC, and an increase in HDL and VLDL (*p* < 0.05). All secondary outcomes (self-care, attitude, knowledge, and empowerment) presented better scores after the intervention with IG, but attitude and empowerment scores also showed improvement for CG in Tf (Table 2).

The average of ΔHbA1c (percentage variation between Tf and Ti) for the control and intervention groups were 3.93 and −5.13, respectively. The difference between the ΔHbA1c of the two groups was considered statistically significant (*p* = 0.029).

**Fig. 3:**
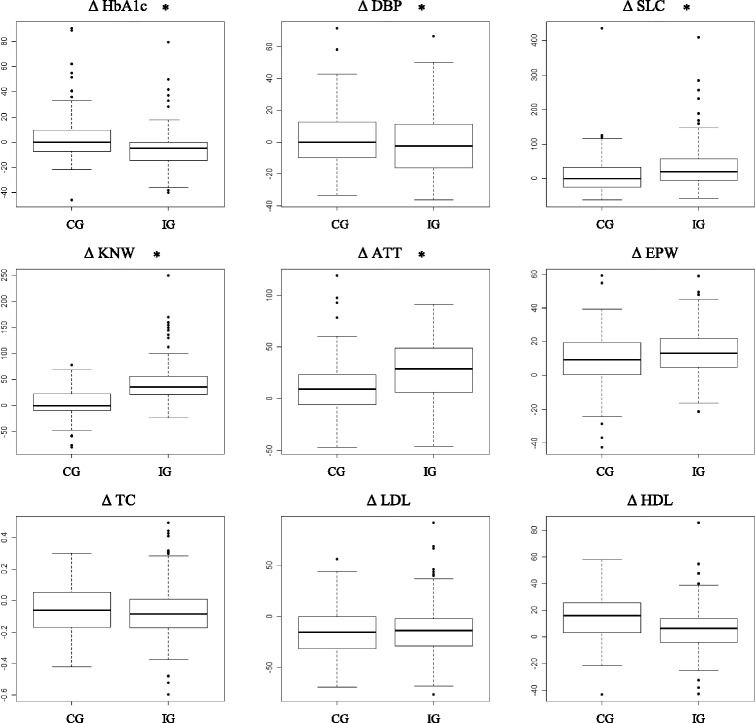
Percentual variations (∆-delta symbol) in measurements between Tf and Ti fir IG and CG. Statistically significant differences between means for IG and CG are marked with * (*p* <0.05). DG: control group; IG: intervention group; HbA1c: glycated hemoglobin; DBP: diastolic blood pressure; SLC: self-care for type 2 diabetes; KNW; knowledge for type 2 diabetes; ATT: attitude for type 2 diabetes; EPW: empowerment for type 2 diabetes; TC; total cholesterol; LDL: low density lipoprotien; HDL: high density lipoprotein

Participants in IG exhibited a greater reduction in the percentage of HbA1c and DBP, and a greater percentage increase in the scores for selfcare, knowledge and attitudes in comparison to individuals in CG (*p* < 0.05) (Fig. 3).

## Discussion

The present study showed favorable results for metabolic control of diabetes mellitus from the empowerment program applied over 12 months. As for the socio-demographic data, our study had young participants, that is, with a lower age for diabetes when compared to other studies [5, 12, 27]. Educational levels were predominantly low, which may reflect the reality of the adult Brazilian population. There was also a lesser presence of men, perhaps because women value self-care more and consequently were more willing to participate in the program [28]. Most of the participants lived with a companion and some studies show that family support is a favorable factor for health care [11, 29].

Appropriate and continuous responses in metabolic control can be hampered by the association of socioeconomic factors such as low education levels with the difficulty health services have providing education for self-care in order to generate sustainable behavior changes with ongoing support that can combine a healthy lifestyle with emotional and physical balance [8]. Still with regard to socio-demographic variables, it is important to emphasize that there was homogeneity between the two groups at the baseline.

Values for HbA1c decreased significantly between Tf and Ti, showing that the empowerment program had a positive effect in reducing this variable, which is important for monitoring diabetes. Clinical trials involving educational programs in other countries also have shown a reduction in HbA1c [5, 12]. Notably, a study by a group of researchers in Italy using the same measuring instruments and protocol used in this study also found positive results [8]. However, prior to this study no studies were identified in Brazil that showed a decrease in this variable after applying an educational intervention. A study conducted in Taiwan showed no significant changes in the mean HbA1c associated with a self-care education program [30]. In addition to the drop in HbA1c in IG, comparison of the matched differences between Tf and Ti between the two groups (IG and CG) also showed a significant decrease, reinforcing the favorable results of the empowerment program.

The lipid profile and DBP also showed significant improvement, with reduction of TC and LDL and an increase in HDL, with the exception of TGL. Control of lipid levels and blood pressure is indispensable for users with diabetes, considering the increased risk for cardiovascular complications they face, as well as arterial insufficiency, nephropathy, and retinopathy, among other conditions [2]. The anthropometric variables BMI and WC, as well as SBP levels, did not show a significant decrease. Studies also had relatively minor impacts in the control of blood pressure and anthropometric data, possibly due to the stringent targets established specifically for diabetes in the users [8, 27].

As for self-care and knowledge about diabetes mellitus, IG showed significant improvements in behavior with positive effects, such as eating habits related to reducing sugar and carbohydrates, and increasing fiber, meal frequency, and physical activity. Studies that used scales for self-care and knowledge about diabetes also showed favorable changes [12, 14, 31]. The empowerment program encouraged participants to make autonomous decisions using knowledge about their self-care behaviors. CG presented worse scores at Tf for self-care and knowledge of diabetes, despite receiving routine care from their respective health units.

The results for attitude and empowerment scale for diabetes presented better scores at the final period in both groups, but after considering the effect of the experiment (the difference between the values at Tf and Ti) it was observed that IG had better scores than CG for attitude. Participants with higher scores on the attitude survey are theoretically more likely to accept their diabetes, which can maximize their chances of self-care for this condition [32].

The empowerment scale is widely used in various randomized clinical studies involving self-management programs for diabetes associated with decreased HbA1c and better scores in self-care and psychological well-being after interventions [8, 12]. It should be noted that not all studies indicate an association between elevating the level of empowerment of users with diabetes and satisfactory biochemical parameters [33].

The results of the instruments used in the study showed the need for behavior change in order to reduce HbA1c. They also helped to identify how users with diabetes deal with emotional and behavioral aspects linked to this chronic condition [9].

The relevance of the results arises from the scarcity of interventions that promote user empowerment for self-care of diabetes in Brazil, beyond the need to incorporate more effective measures for diabetes control in the country [8]. In this regard, it was observed that participating in the program improved biochemical parameters, attitudes, and behaviors in the face of this condition. Studies show that metabolic control of diabetes is the result of self-care in the user, in that the individual is able to manage their daily control with behavior changes based on healthy lifestyle habits. They also show that the use of protocols to guide educational practices in a co-responsible manner maximizes the program's activities in developing self-care [5, 10, 34].

The homogeneity between CG and IG at baseline reinforced that the interventions carried out over 12 months yielded satisfactory responses related to behavior change and biochemical and anthropometric parameters.

Among the limitations of the study were, first, telephone monitoring that may have reduced evasion in IG, since the CG has not been monitored. At the same time it may have encouraged the subjects to achieve the goals they proposed. In any case, this bias reinforces what the literature has reported throughout history—that education also takes place through repetition, in other words, this type of empowerment program for chronic conditions needs to be perpetuated by the health team serving the area where the users with diabetes are located. Second, medication adjustments are inevitable in managing diabetes mellitus, which can influence the findings of the study. However, the objective of this study was not to evaluate this relationship, in either group. Both groups received secondary treatment from their health teams. Third, it is known that the charisma and the skills that researchers use to guide the groups directly influence increased or decreased user participation in achieving goals. Even with a protocol to be followed, it would be worthwhile in the future to compare the results from empowerment programs led by trained and untrained professionals.

## Conclusion

The empowerment program based on individualized goals for changing psychosocial, behavioral, and clinical aspects was effective in improving self-care practices and metabolic control of diabetes mellitus in Brazilian users.

## Appendix

**Table 3:** Analysis of the losses in IG after randomization

Variable	Mean ± SD or Median (Min-Max) or N (%)		*p*
	Left (*n* = 35)	Remained (*n* = 127)	
Age (Mean ± SD)	55.1 ± 11.97	58.1 ± 9.22	0.12*
HbA1c (Median (Min/Max))	7.9 (5.6–12.3)	7.7 (5.3–13.5)	0.50**
IMC (Median (Min/Max))	29.4 (21.9–41.6)	29.8 (19.1–52.4)	0.89**
Sex (N (%))			
Male	15 (42.9)	42 (33.1)	0.28***
Female	20 (57.1)	85 (66.9)	
Education Level (N (%))			
Incomplete elementary school	26 (74.3)	90 (70.9)	0.69***
Elementary school complete to post-graduate	9 (25.7)	37 (29.1)	

* Simple Student *t*-test, **Mann–Whitney, ***Chi-squared

CG: Control group; IG: Intervention group

**Table 4:** Analysis of the losses in CG after randomization

Variable	Mean ± SD or Median (Min-Max) or N (%)		*p*
	Left (*n* = 11)	Remained (*n* = 111)	
Age (Mean ± SD)	57.2 ± 5.15	57,5 ± 9.69	0.88*
HbA1c (Median (Min/Max))	7.2 (6.3–8.4)	7.4 (5–14.4)	0.70**
IMC (Median (Min/Max))	29.3 (26.6–35.9)	29.4 (17.1–46.3)	0.97**
Sex (N (%))			
Male	4 (36.4)	38 (34.2)	0.89***
Female	7 (63.6)	73 (65.8)	
Education Level (N (%))			
Incomplete elementary school	6 (54.6)	73 (65.8)	0.18***
Elementary school complete to post-graduate	5 (45.4)	38 (34.2)	

* Simple Student *t*-test, **Mann–Whitney, ***Chi-squared

CG: Control group; IG: Intervention group

## Abbreviations

DM2: Type 2 diabetes mellitus
CG: Control Group
IG: Intervention group
HbA1c: Glycated hemoglobin
TC: Total Cholesterol
HDL: High density lipoprotein
LDL: Low density lipoprotein
TGL: Triglycerides
BMI: Body mass index
SBP and DBP: Systolic and diastolic blood pressure
WC: Waist circumference
SLC: Self-care for DM2
KNW: Knowledge for DM2
ATT: Attitude for DM2
EPW: Empowerment for DM2
